# Modeling and Simulation to Support Phase Ib/IIa Dose Selection for WBP216, A Long Half-Life Fully Human Monoclonal Antibody Against Interleukin-6

**DOI:** 10.3389/fphar.2021.617265

**Published:** 2021-02-18

**Authors:** Xiange Tang, Xiaofeng Zeng, Xiaoduo Guan, Rui Chen, Pei Hu

**Affiliations:** ^1^ Clinical Pharmacology Research Center, Peking Union Medical College Hospital, Chinese Academy of Medical Sciences and Peking Union Medical College, Beijing, China; ^2^ Key Laboratory of Clinical PK and PD Investigation for Innovative Drugs, Beijing, China; ^3^ Department of Rheumatology, Peking Union Medical College Hospital, Chinese Academy of Medical Science and Peking Union Medical College, Beijing, China

**Keywords:** population pharmacokinetic, population pharmacodynamic, antibody, rheumatoid arthritis, dose optimization, model

## Abstract

WBP216 is an innovative IL-6 antibody, presenting high affinity to IL-6 and a long half-life (40–60 days). To optimize the dosage regimen for future clinical trials, pharmacokinetics (PK) and pharmacodynamics (PD) of WBP216 would be firstly characterized in Chinese rheumatoid arthritis (RA) patients. PK, CRP and DAS28 data of WBP216 were collected from 26 RA patients in a single ascending dose study. Non-linear mixed effects modeling was used for a population PK/PD analysis. A two-compartment model with a sequential zero-first order absorption and a first order elimination best described PK behavior of WBP216. Apparent systemic clearance was 0.015 L/h, central volume was 8.04 L. CRP as the fast-decreasing endpoint and DAS28 as the slow-reacting endpoint were both fitted well through an indirect response model. The baseline of ALT and free IL-6 were found associated with PK/PD parameters during covariates exploration. Simulation results confirmed that a loading dose regimen either of administration at weeks 0, 2, and 6 or doubling the maintenance dose level, followed by maintenance dosing of 75–150 mg every 8 weeks, was expected to provide a best risk/benefit ratio in future clinical studies. We hope this first PK/PD study of WBP216 in Chinese RA patients will help in the clinical development of WBP216 in future and provide a reference to the dosage optimization of similar antibodies with long half-life.

**Clinical Trial Registration:** CTR20170306

## Introduction

WBP216 is an IgG1 antibody that binds and neutralizes IL-6 and is designed as a biologic anti-rheumatic drug. Rheumatoid arthritis (RA) is featured by progressive articular disability, systemic inflammation, and high morbidity, which stem from a complex interaction between various inflammatory cells and cytokines (McInnes and Schett, 2011; Smolen et al., 2016). Biological therapies are recommended for treating RA refractory to synthetic chemical drugs (Newsome, 2002). IL-6 is one of major pro-inflammatory cytokines involved in RA pathogenesis. Inhibition of the IL-6 signaling pathway helps to reduce inflammation and pain in patients with RA (Ohsugi and Kishimoto, 2008; Raimondo et al., 2017). Tocilizumab, as an anti interleukin 6 receptor (IL-6R) monoclonal antibody, is launched in 2009 for the indication of RA, whose successful clinical use has proved the key role of IL-6 in RA pathogenesis (Choy et al., 2002).

WBP216 is a fully humanized monoclonal antibody that has showed its significant efficacy in treatment of RA in phase Ⅰ trial. WBP216 has a strong affinity for interleukin 6 (IL-6), with an affinity constant in the picomolar range. WBP216 prevents the interaction of IL-6 and its receptor, thus reducing pro-inflammatory activity. Unlike regular IgG1 antibodies, WBP216 includes YTE mutations in the fragment crystallization (Fc). These mutations increase the ability of the Fc fragment to bind the neonatal Fc receptor (FcRn) (Oganesyan et al., 2009), which protect WBP216 from intracellular degradation, and thereby extending its half-life to >40 days and bringing some different pharmacokinetic (PK) characters. Thus, WBP216 has the potential to relieve RA effectively and for a sustained period.

Due to the blockade of IL-6 signaling, WBP216 can directly inhibit the hepatic production of C-reactive protein (CRP) directly (Bastida et al., 2019) and the erythrocyte sedimentation rate (ESR). Thus, the levels of inflammatory markers (CRP and ESR) rapidly decrease after the initiation of IL-6 blocker treatment, even prior to any improvement in swollen or tender joint counts. Therefore CRP alone is not sufficient to assess efficacy. According to the American College of Rheumatology (ACR) response criteria, the disease activity score based on 28 joint (DAS28) in its two versions employing ESR or CRP are most frequently used in clinical trials and also in clinical practice. DAS28 includes assessment of swollen/tender counts for 28 joints and patient/evaluator physician global assessment, and usually decreases slowly after RA treatments compared to CRP and ESR (Smolen et al., 2003). Hence, rapidly and slowly decreasing pharmacodynamic (PD) biomarkers should be assessed together to comprehensively evaluate RA medications.

Population PK/PD modeling and simulation has proven to be a useful approach in facilitating drug development (Budha et al., 2015). We report here the population PK/PD analysis of WBP216 PK and serum CRP and DAS28-ESR data in RA patients from phase Ⅰa, and model-based simulation results. The goal of this modeling and simulation is to optimize dose levels, dosing intervals and loading dose regimens for such a long half-life antibody in Phase Ⅰb/IIa, a multiple ascending dose (MAD) study, which will help to improve the drug development efficiency. Moreover, a population PK/PD analysis in this phase can lead to a better understanding of PK/PD characteristics of WBP216. PK/PD data obtained directly from RA patients instead of healthy volunteers allows to recommend more accurate dosing regimens that can provide significant CRP and DAS28-ESR reduction while minimizing the frequency of subcutaneous injections.

Therefore, we aimed to establish a population PK/PD model of WBP216 using phase Ia PK/PD data in RA patients and then use the model to make phase Ib/IIa dose regimen decision in this study. To our knowledge, this is the first report of the PK/PD of WBP216 in Chinese RA population. Our study will provide valuable information for the development and application of WBP216 in clinic.

## Methods

### Study Population and Data

Briefly, phase Ⅰa (CTR20170306) was a multi-center, randomized, double-blinded, single ascending dose study of WBP216 in RA patients. The study protocol was designed in full compliance with Good Clinical Practice and the Declaration of Helsinki and approved by the independent ethics committee of Peking Union Medical College Hospital and Beijing hospital (Beijing, China). Subjects eligible to take part in this study were RA patients diagnosed over 6 months, treated with basic RA medications (e.g., methotrexate and leflunomide) stably for at least 28 days, aged from 18 to 70 years and with a body mass index (BMI) of 19–30 kg/m^2^. All subjects signed the Informed Consent Form before their participation.

A total of 36 subjects were enrolled into 5 dose cohorts (10, 30, 75, 150 or 300 mg) respectively and randomized to receive WBP216 or placebo subcutaneously, with 3 subjects receiving active WBP216 and 1 subject receiving placebo in the lowest dose cohort (i.e., 10 mg) and with 6 subjects receiving WBP216 and 2 subjects receiving placebo in other each dose group. Serum samples were collected at pre-dose and 0.083, 0.5, 1, 3, 7, 14, 21, 28, 42, 56, 84, 112, 168 days post-dose to obtain total WBP216 concentration and CRP data. Total WBP216 was assayed by validated methods whose linear calibration ranges were 39.1–10,000 ng/ml for WBP216. The CRP and ESR measurements were conducted by the clinical laboratory center of the hospitals. The LLOQ for CRP and ESR are 0.01 mg/L and 1 mm/h, respectively. DAS28-ESR and ACR scores were estimated by doctors at baseline and on days 7, 28, 56, 84, 112, 140 and 168, with 112/140 days measurements only for the 75–300 mg groups. If any anti-drug antibody (ADA) measurement was positive in any subject during the trial period, this subject was described as ADA positive.

### Characterization of WBP216 PK Properties

The YTE mutations in the Fc fragment of WBP216 may result in non-standard extended PK properties, which is one of our study focus. In order to explore the PK characteristics, PK parameters were firstly calculated based on individual plasma concentration-time-profiles using non-compartment analysis (NCA) by validated Phoenix WinNonlin version 8.1 software (Pharsight Corporation, Mountain View, CA, USA). PK parameters through NCA were then analyzed and explored using WinNonlin or PRISM (GraphPad 8.0.1, San Diego, CA, USA). A power model (Eq. 1) proposed by Gough et al. was applied to assess the dose proportionality (Kevin Gough, 1995).

log(Y)=μ+β×log(dose) (1)

in which *Y* denotes PK parameters such as area under curve (AUC) or maximum concentration (C_max_). This approach assumes that the underlying relationship between log(*Y*) and log(dose) is linear. *β* = 1 indicates total dose proportionality. In our study, a less stringent criterion was used (Hummel et al., 2009), given the small sample size in phase Ⅰ across multiple dose groups. The estimate of *β* together with CI falling completely within the range of [0.5–2] was quantified as dose proportionality.

### Population PK and PD Model Development

The relationship between drug exposure and response was evaluated using nonlinear mixed effects models (Phoenix NLME, version 8.1, Certara). First-order conditional estimation, extended least squares method (FOCE-ELS) was used to estimate pop PK/PD model parameters. The final structural model was determined by the objective function value (OFV) and Akaike information criterion (AIC). A sequential modeling strategy was used for fitting the models to the phase Ⅰa data. The population PK model was first developed and then the individual post hoc parameters from the final PK model were used to predict the individual WBP216 concentrations to drive the drug effect on CRP or DAS28 time profiles using appropriate PD models.

We tried one, two or three-compartment PK model with a first order, saturate or sequential zero-first order absorption compartment. The elimination phase was also analyzed by fitting to first order, saturate elimination or target-mediated-drug-disposition (TMDD) models. Based on the mechanism of action, an indirect-response model was chosen as a starting point for PD model development for CRP or DAS28. A linear model, an E_max_ model or a sigmoidal E_max_ model (Hill equation) were applied to characterize the relationship between WBP216 serum concentrations and those disease activity measures.

Inter-individual variability (IIV) was assumed to follow a log-normal distribution and was described using exponential model (Eq. 2).

$$P_{ij}=\theta_{i}\times \;e^{\eta ij}$$ (2)

where *P*_*ij*_ represents the individual value of the parameter for the *i*th parameter in the *j*th individual, *θ*_*i*_ depicts the population typical value for the *i*th parameter, and *ηij* represents random effect in *j*th individual sampled from a normal distribution with a mean of zero and variance of *ω* ^2^.

The residual unexplained variability of WBP216 concentration and PD observation was described by a proportional or additive error model, respectively (Eqs 3, 4).

$$Y_{\text{obs}}\;=\;Y_{\text{pred}}\times \left(1+\varepsilon_{1}\right)$$ (3)

$$Y_{\text{obs}}=Y_{\text{pred}}+\varepsilon_{2}$$ (4)

where *Y*_*obs*_ and *Y*_*pred*_ are the observed and predicted serum concentration in plasma or disease activity measures. And *ε*_*1*_ is the proportional and *ε*_*2*_ is the additive component of the residual error model, respectively. Both of *ε*_*1*_ and *ε*_*2*_ are assumed to be normally distributed in the range of (0, *σ* ^2^).

After collinearity diagnostics and correlation analysis, possible covariates including weight, age, sex, baseline serum albumin (ALB), alanine amiotransferase (ALT), creatinine clearance (CLcr), total bilirubin (TBIL), free IL-6, ADA (negative or positive) etc. were tested on both PK and PD parameters. Continuous covariates were described using the power function and categorical covariate were modeled by exponential function, see Eqs 5 and 6.

$${\text{Effect}}_{i}={\left({\text{Cov}}_{ij}/{\text{Cov}}_{\text{median}}\right)}^{\theta \mathrm{cov}i}$$ (5)

$${\text{Effect}}_{i}=e^{\text{Cov}ij\cdot \theta \mathrm{cov}i}$$ (6)

where *Effect*_*i*_ is the multiplicative factor for covariate *i*, *Cov*_*ij*_ is the covariate value for individual *j*, *Cov*_*median*_ is the median covariate value, and *θcov*_*i*_ is the exponent or parameter for covariate *i* model.

Potential covariates were incorporated into the base model one by one using stepwise forward inclusion. When OFV decreased by 6.63 (at *p* < 0.01), the covariate was selected for inclusion to develop a full model, followed by the backward elimination. The covariates were subtracted one at a time in a stepwise manner as well once OFV increased above 10.83 (at *p* < 0.001, df = 1), until all remaining covariates in model were statistically significant.

### Model Evaluation

During the process of models building, the goodness of fit (GOF) of different models was compared on the basis of OFV and AIC. GOF was graphically evaluated by inspecting plots of the individual or population predicted vs. observed values, and plots of the conditionally weighted residuals (CWRES) vs. population predictions or time.

Models were also validated internally using prediction-corrected visual predictive checks (pcVPC) as well (Bergstrand et al., 2011). On the basis of 1,000 times pcVPC simulation, the 90% prediction interval (PI) was compared with the 90% interval of the prediction-corrected observations. Bootstrap analysis was also performed for the final model along with a total of 500 data sets resampling randomly from the original data set (Ette, 1997). We reported the calculated 90% confidence interval (CI) of model parameters from successfully minimized runs.

### Simulations for Phase Ib Dose Selection

Simulation was conducted in Phoenix NLME (version 8.1, Certara, USA) based on a Monte-Carlo simulation approach. Up to 100 Phase Ib trials were simulated using the uncertainty distribution in parameter estimates. 27 patients in each simulated trial were simulated using the IIV log-normal distribution in both PK and PD parameters. The distribution of covariates still leveraged those of phase Ⅰa data set. Serum CRP levels and DAS28 were simulated for a range of maintenance doses (30–300 mg) under three different dosing frequencies: every 4 weeks (Q4W), every 8 weeks (Q8W), and every 12 weeks (Q12W). The duration of drug effect was simulated up to week 72 with weekly virtual PD sampling. It was assumed that the PK/PD relationship based on the Phase Ⅰ study lasting 24weeks could be extrapolated to longer term studies.

Tocilizumab has proven its successful clinical efficacy, so it was used as the reference for comparison of clinical endpoints. Since mean CRP is decreased by around 90% and mean DAS28 could be reduced by over 56.5% using the recommended dosage of tocilizumab (ACTEMRA^®^ HIGHLIGHTS OF PRESCRIBING INFORMATION; U.S. Food and Drug Administration), ∆CRP≥90% and ∆DAS28 ≥ 56.5% were set as our target efficacy to optimize WBP216 phase Ⅰb maintenance dose levels and dosing frequencies.

WBP216 would take a long time to achieve steady state exposure and efficacy because of its slow elimination rate constants. Because RA patients would require a rapid pain relief, a loading regimen of WBP216 would be necessary. Hence, we simulated two categories of loading regimen to achieve steady state exposure: Firstly, WBP216 was given in a more intensive frequency at an initial three-administrations, including 0–4–8th week, 0–2–4th week or 0–2–6th week; The second simulation used a loading dose, that doubled the confirmed maintenance dose level.

## Results

### Clinical Data Summary

Up to 36 RA patients took active medicine and placebo in a ratio of 3:1, respectively, wherein 27 patients received WBP216. One of the subjects in the 75 mg group showed a huge fluctuation of CRP level after 21 days from administration, very different from other subjects. The CWRES of there CRP samples were also greater than six during CRP model exploration. This subject was considered as an outlier and excluded from this PK/PD model analysis. Descriptive statistics (baseline values) of potential covariates of 26 patients tested in the PK/PD analysis were summarized in Table 1. A total of 391 PK samples were obtained during the phase Ⅰa and PD data consisted of 384 CRP samples and 241 DAS28 samples. Since fewer than 10% of PK samples were below the lower limit of quantification (LLOQ), they were handled by M1 method (Keizer et al., 2015). PK concentrations whose corresponding ADA was positive were all above the LLOQ, so they were not discarded.

**TABLE 1:** Descriptive statistics of the demographic characteristics, laboratory data and disease activity (baseline values) of the RA patients included in the population PK/PD model (*n* = 26).

Covariates	Value (Mean ± SD)
Demographic	
Sex-female, *n* (%)	23 (88.5%)
Age (years)	49.7 ± 10.1
Weight (kg)	61.5 ± 7.9
Height (cm)	161.0 ± 7.0
Laboratory data	
Albumin (ALB, g/L)	40.2 ± 3.4
Alanine transaminase (ALT, U/L)	14.1 ± 7.0
Total bilirubin (TBIL, μmol/L)	10.8± 3.9
Creatinine clearance (CLcr, ml/min)	104.6 ± 31.8
Positive ADA, n (%)	3 (11.5%)
Free IL-6 (baseline, pg/mL)	54.8 ± 87.9
CRP (baseline, mg/L)	14.7± 19.9
Disease activity	
DAS28 (baseline)	5.3 ± 0.9

### Inspection of WBP216 PK Properties

**FIGURE 1:**
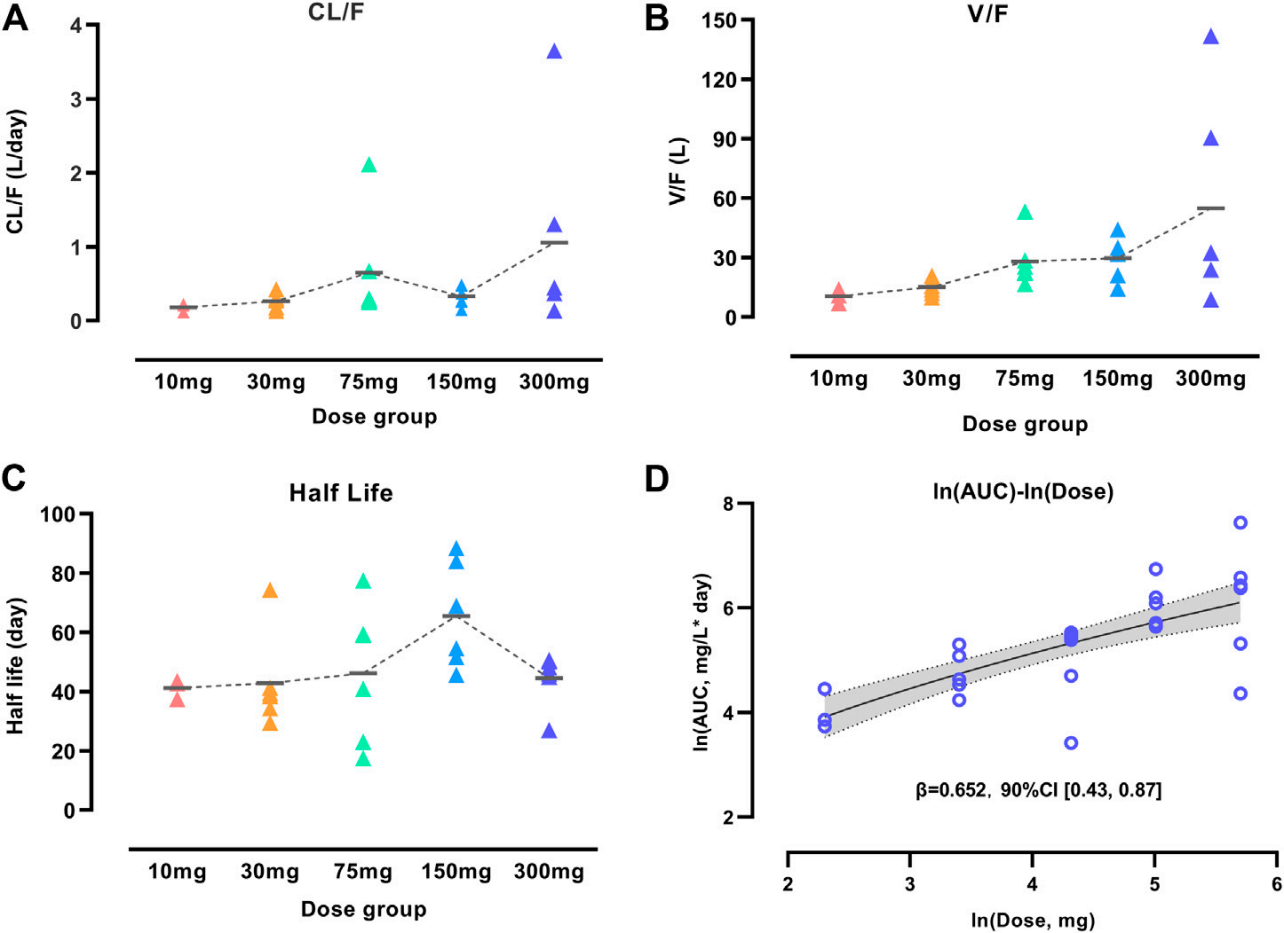
Characterization of WBP216 pharmacokinetic properties. Apparent clearance vs. dose groups **(A)**, Apparent volume vs. dose groups **(B)**, Comparison of half-life in different dose groups **(C)**. The short horizontal line represents the mean value of CL/F, V/F and t1/2, respectively, in five dose groups. Dose proportionality assessment using power model **(D)**. The dashed lines are the connection of those mean value; The solid line denotes the expected mean value and the shaded region denotes the 90% confidence interval.

We performed an NCA analysis for different dose groups before population PK model development to understand the PK properties fully because of the unique YTE mutations in WBP216. Figure 1 showed that mean apparent clearance (CL/*F*) tended to increase with increasing dose, contrary to the clearance change pattern of TMDD, which generally has a high clearance in lower dose groups (Mager, 2006). Large variability of CL/*F* among individuals was observed in both 75 and 300 mg dose groups. High individual variability of apparent volume (V/*F*) was also observed in higher dose levels (75–300 mg). Like CL/*F*, V/*F* presented an increasing trend over dose levels. The phenomenon of CL/*F* and V/*F* changing with dose levels was speculated to result from the changes of either the actual increased CL and V or decreased bioavailability (*F*). To clarify the real reason, the half-life of WBP216 was analyzed. Half-life (*t*_1/2_ = 0.693*V/CL) is considered not to be impacted by *F*. In this analysis of half-life, the value of *t*_1/2_ distributed evenly in five dose groups, and the mean *t*_1/2_ remained almost the same in different groups, except for the 150 mg group, likely caused by lower CL/*F* and higher V/*F* value in this group. Those changing trends of CL/*F*, V/*F* and *t*_1/2_ suggested that the decrease of *F* with doses was probably the cause of the increase of CL/*F* and V/*F*. Mean *t*_1/2_ is 40–60 days, indicating the potential for a long dosing interval in therapeutic use. The power model fitted ln (AUC) over ln (dose) data well (Figure 1D). Parameter *β* was calculated as 0.652 with 90% confidence interval (CI), [0.43, 0.87], part of which fell outside the lower limit of prespecified range [0.5, 2] (Hummel et al., 2009). This dose-dependent study indicated little lower than proportional increases in exposure (AUC).

### Population PK/PD Model Development

**FIGURE 2:**
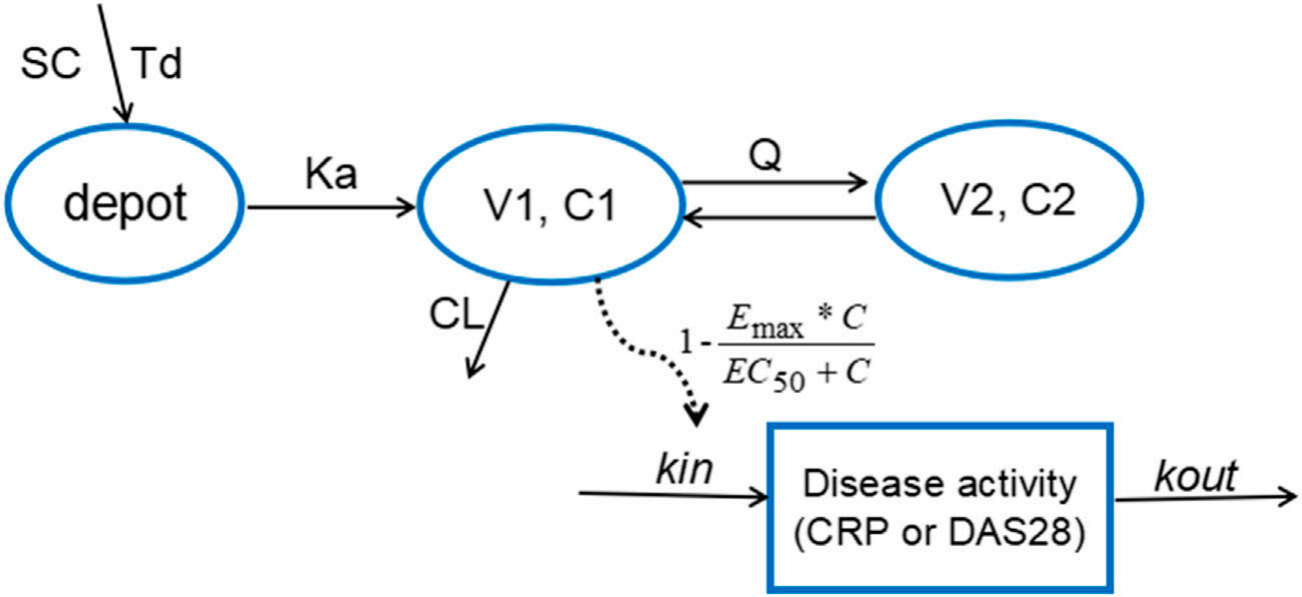
The proposed PK/PD model structure. Parameters are abbreviated as follows: SC = subcutaneous injection; Td = time of zero order release; Ka = first order absorption rate; C1 = concentration in central compartment; V1 = central volume; CL = clearance from central compartment; C2 = concentration in peripheral compartment; V2 = volume of peripheral compartment; Q = clearance between central and peripheral compartments; E_max_ = maximum drug efficacy; EC_50_ = concentration needed for 50% of E_max_; K_in_ = zero order rate constant for response production; K_out_ = first order rate for response loss.

According to AIC value and goodness of fit (GOF) plots, the selected final proposed PK/PD model structure is shown in Figure 2. The WBP216 serum concentrations were best described by a two-compartment PK model with sequential zero-first order absorption and first order elimination (see Eqs 7–9). An indirect-response model with an drug E_max_ inhibition of the CRP or DAS28 zero order rate production constant (K_in_) best described the disease measures (see Eqs 10, 11). Residual variability was characterized by a proportional error model for serum concentration, CRP and DAS28. All PK/PD model parameters are summarized in Table 2.

$$\frac{dA_{\text{depot}}}{dt}=\frac{\text{Dose}}{Td}-\text{ka}\times A_{\text{depot}}\left(t=0,\;A_{\text{depot}}=0\right)$$ (7)

$$\frac{dA1}{dt}=ka\times A_{\text{depot}}-CL/F\times C1-Q/F\times \left(C1-C2\right)\;\left(t=0,A1=0\right)$$ (8)

$$\frac{dA2}{dt}=Q/F\times \left(C1-C2\right)\;\left(t=0,\;A2=0\right)$$ (9)

$$\frac{d\text{CRP}}{dt}=K_{\text{in,CRP}}\times \left(1-\frac{E_{\max,CRP}\times C1}{EC_{50,CRP}+C1}\right)-K_{\text{out,CRP}}\times \text{CRP}$$ (10)

$$\frac{\mathrm{dDAS}28}{dt}=K_{\text{in},\text{DAS}28}\times \left(1-\frac{E_{\max,DAS28}\times C1}{EC_{50,DAS28}+C1}\right)-K_{\text{out,DAS}28}\times \text{DAS}28$$ (11)

Wherein *A*_depot_, A1, A2 representing WBP216 amounts in absorption depot, systemic central and peripheral compartments respectively, were equal to zero when *t* = 0. C1 and C2 were the concentration in central and peripheral compartment, respectively and equal to zero before dosing. Response was CRP or DAS28, and was equal to its baseline value before drug administration.

**TABLE 2:** Summary of final population pharmacokinetic and pharmcodynamic model parameters in rheumatoid arthritis patients.

Parameters	Definition	Estimated value (RSE%)	IIV(RSE%)	Bootstrap of estimates median (90%CI)	Bootstrap of IIV median (90%CI)	Shrinkage
PK model						
Ka (1/hr)	Subcutaneous absorption rate constant	0.007 (20.8)	54.1% (13.3)	0.007 (0.005, 0.01)	50.7% (24.6%, 60.9%)	14.3%
Td (hr)	Release time of zero order rate	2.183 (29.0)	128.4% (21)	2.173 (1.221, 3.246)	124.5% (73.5%, 194.8%)	16.9%
CL/F ( L/h)	Clearance from central compartment	0.015 (12.4)	52.7% (13.2)	0.014 (0.011, 0.018)	50.3% (37.5%, 61.0%)	−0.4%
V1/F (L)	Volume of central compartment	8.039 (30.9)	117.1% (11.1)	8.268 (5.024, 14.24)	112.4% 90.5%, 132.4%)	0.4%
V2/F (L)	Volume of peripheral compartment	10.298 (11.6)	48.2% (27.0)	10.141 (7.27, 12.124)	45.8% (30.9%, 69.9%)	18.1%
Q/F (L/h)	Clearance between central and peripheral compartment	0.062 (20.9)	NE	0.060 (0.048, 0.120)	NA	NA
*θ*_ALT∼CL_	Covariate about ALT on CL	−0.833 (30.9)	NA	−0.830 (−1.113, −0.196)	NA	NA
σ_PK_	Proportional error for serum concentration	0.119 (8.9)	NA	0.118 (0.102, 0.137)	NA	NA
CRP model						
K_in,CRP_ (mg/(L*hr))	Zero-order constant for response production	0.185 (19.7)	93.2% (14.3)	0.187 (0.071, 0.323)	86.9% (66.0%, 107.5%)	4.5%
K_out,CRP_ (1/hr)	First-order rate constant for response loss	0.026 (4.6)	NE	0.026 (0.024, 0.029)	NA	NA
EC_50,CRP_ (ug/L)	The concentration to achieve 50% E_max,CRP_	194.37 (24.7)	113.0% (16.6)	202.887 (124.151, 578.5)	107.9% (79.8%, 138.8%)	8.6%
E_max,CRP_	The maximum effect of drug	1 (fixed)	NA	NA	NA	NA
*Θ*_BaseFreeIL-6∼kin_	Covariate about baseline of free IL-6 on K_in,CRP_	0.695 (14.9)	NA	0.710 (0.519, 0.855)	NA	NA
*Θ*_BaseFreeIL-6∼EC50_	Covariate about baseline of free IL-6 on EC_50,CRP_	−0.772 (17.6)	NA	−0.773 (−1.021, −0.557)	NA	NA
σ_CRP_	Proportional error for CRP	0.523 (13.1)	NA	0.524 (0.422, 0.640)	NA	NA
DAS28 model						
K_in,DAS28_ (1/hr)	Zero-order constant for response production	0.003 (11.1)	NE	0.003 (0.002, 0.004)	NA	NA
K_out,DAS28_ (1/hr)	First-order rate constant for response loss	0.0006 (11.5)	17.0% (12.8)	0.0006 (0.0004, 0.0009)	16.5% (11.9%, 21.5%)	8.1%
EC_50,DAS28_ (ug/L)	The concentration to achieve 50% E_max,DAS28_	1,576.3 (15.6)	95.5% (13.5)	1,602.1 (1,014.4, 2,513.9)	92.2% (55.9%, 120%)	15.4%
E_max,DAS28_	The maximum effect of drug	1 (fixed)	NA	NA	NA	NA
σ_DAS28_	Proportional error for DAS28	0.18 (12.0)	NA	0.177 (0.143, 0.217)	NA	NA

RSE%: relative standard errors; IIV: Inter-individual variability; 90% CI: 90% confidence interval; NE: not estimate; NA: not applicable.

The final PK model fitted the observed concentration data better by assuming that after WBP216 was subcutaneously administered, drug was released from the injection site at a zero order rate to a depot compartment, and was then absorbed to a central compartment. Td is the period of zero order release and Ka denotes the first order absorption rate. The reason of selecting such a more complex absorption model will be discussed later. Based on the mechanism of action, WBP216 neutralizes IL-6 and inhibits CRP production, and further slow down disease progression, so it is plausible to set the drug inhibition on response production rate, K_in_ (Sharma and Jusko, 1998). As shown in Table 2, the relative standard errors (RSE%) for almost all fixed-effect parameters were ≤30.9%. The uncertainties for random-effect parameters were <27%. Overall, the precision of parameters estimates was acceptable. Parameters showed various inter-individual variability, ranging from 17 to 128.4%. *E*_max_ for CRP was set to 1 since its value was always very close to 1 in all tested runs. E_max_ for DAS28 was also fixed to 1, thus leading to a straightforward convergence of the model. All applicable shrinkage was below 18.1%, which was smaller than reported cut-off value 30% and could assure accurate IIV estimates and avoidance of misleading diagnostic plots (Savic and Karlsson, 2009).

The correlation diagnosis chart of various covariates was presented in Supplementary Figure S1. The potential impacts of demographics and laboratory data baseline on PK/PD parameters of WBP216 were tested using a stepwise covariate modeling procedure. Those statistical significant covariate effects were identified and retained in the final model: ALT on CL, baseline free IL-6 on EC_50,CRP_ and K_in,CRP_ (see Eqs 12–14 and Supplementary Table S2). Continuous covariates were described using the power function, centered by the median value. Apparent clearance decreased with increasing ALT with exponent −0.833, which explained around 18% of CL/F inter-individual variability (IIV). Higher baseline free IL-6 levels could result in increased K_in,CRP_ (exponent 0.695), while lead to its EC_50,CRP_ reduction (exponent −0.772). Baseline free IL-6 accounted for 59.2% IIV of K_in,CRP_ and 55.6% IIV of EC_50,CRP_. No covariates were found statistically significant in parameters for DAS28. Covariate associated parameters are also presented in Table 2.

$$\text{CL/}F=\theta_{\text{CL/}F}\times {\left(\frac{\text{ALT}}{11}\right)}^{-0.833}\times e^{\eta \text{CL/F}}$$ (12)

$${\text{K}}_{\text{in,CRP}}=\theta_{\text{Kin,CRP}}\times {\left(\frac{\text{BaseFreeIL-6}}{24.9}\right)}^{0.695}\times e^{\eta_{{\text{K}}_{\text{in,CRP}}}}$$ (13)

$${\text{EC}}_{50\text{,CRP}}=\theta_{{\text{EC}}_{50\text{,CRP}}}\times {\left(\frac{\text{BaseFreeIL-6}}{24.9}\right)}^{-0.772}\times e^{\eta \text{EC50,CRP}}$$ (14)

### Model Diagnosis and Evaluation

**FIGURE 3:**
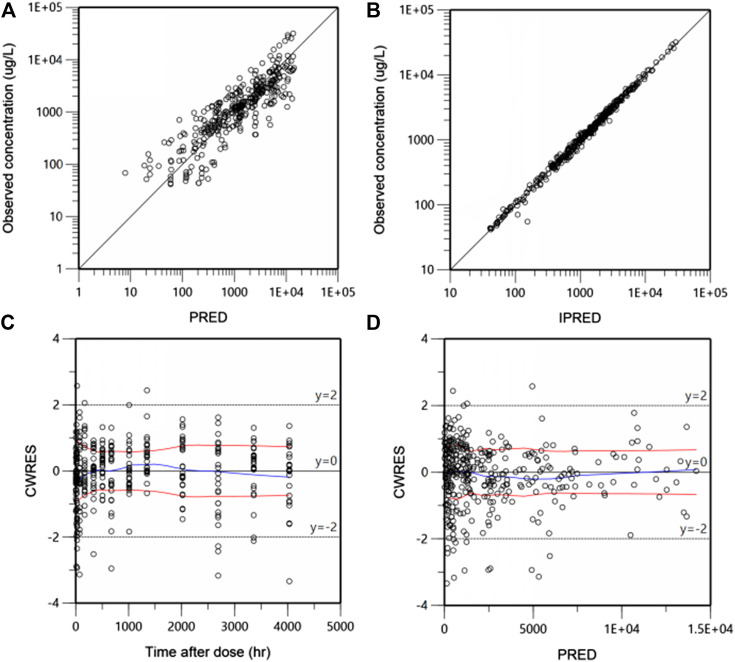
Goodness-of-fit plots for the final population pharmacokinetic (PK) model. Population-predicted concentration vs. observed concentrations (ug/L) **(A)**, Individual-predicted concentration vs. observed concentrations **(B)**, The conditional weighted residuals (CWRES) over time after dose (h) **(C)**, CWRES against population predicted concentration **(D)**. The blue lines are the smoothed LOESS regression lines and the red lines represent LOESS regression to the absolute values of the dependent variable with its negative reflection.

Goodness-of-fit (GOF) plots for the final PK model in serum are shown in Figure 3. Plots of the population- and individual- predicted concentration vs. observed concentrations demonstrate no major bias. The conditionally weighted residuals (CWRES) were symmetrically distributed about zero axis and most points laid within the acceptable range (−2 to 2), suggesting that little to no bias accompanied with concentration or time. The GOF plots for CRP and DAS28 model were presented in Supplementary Figures S2 and S3, respectively, which also performed well in visual diagnostic.

**FIGURE 4:**
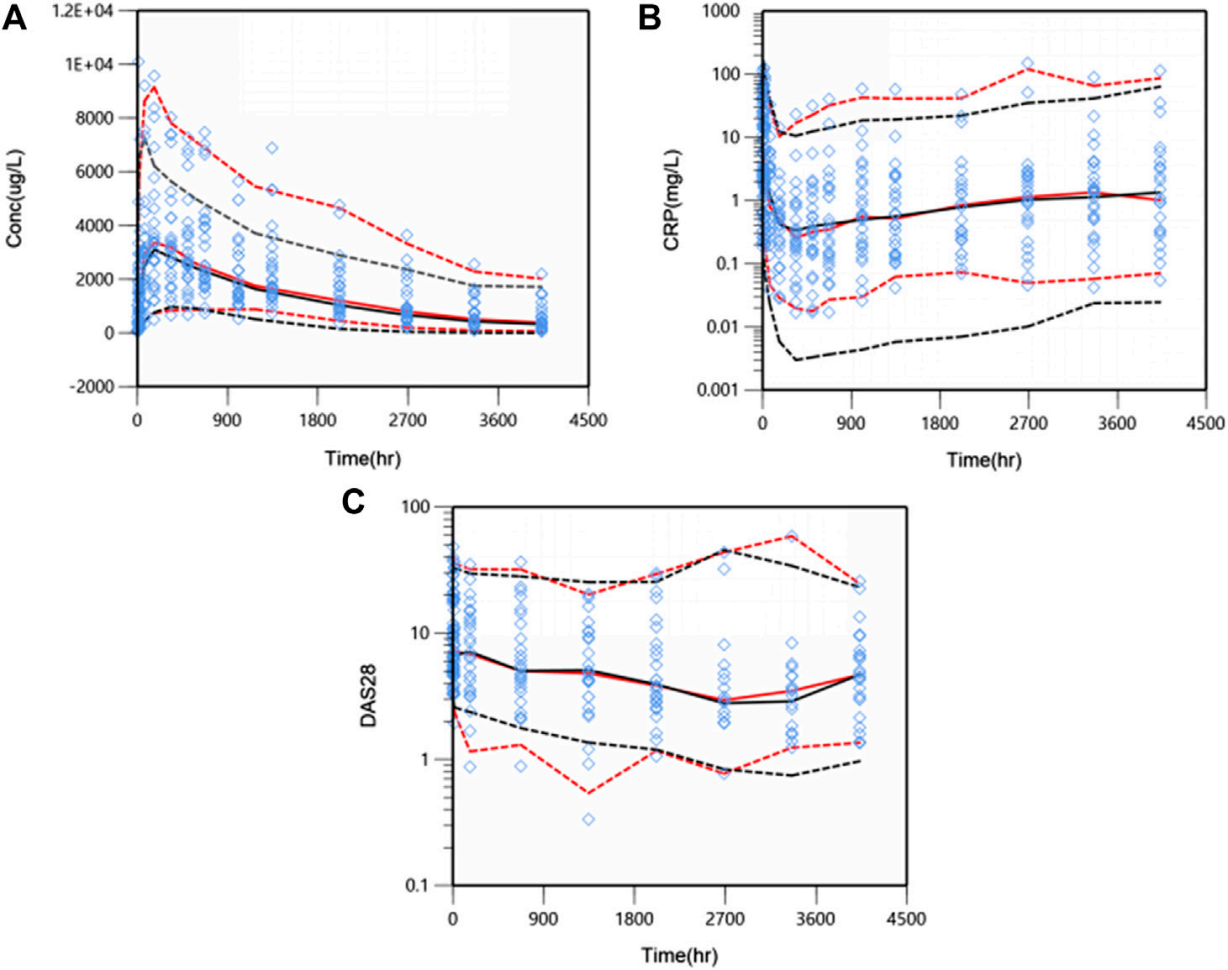
Prediction-corrected visual predictive check (pcVPC) results for PK/PD model. WBP216 serum concentrations vs. time within 24 weeks **(A)**, CRP against time within 24 weeks **(B)**, DAS28 over time within 24 weeks **(C)**. Open diamonds represent observation points. Red dash/solid lines denotes 5th, 50th, and 95th percentiles for prediction-corrected observations; black dash/solid lines are 5th, 50th, and 95th percentiles for prediction, respectively.

The predictive performance was evaluated internally by pcVPC. Plots of pcVPC were presented in Figure 4. We can see that the 5th, 50th and 95th percentiles of prediction-corrected observations and predicted data were fairly consistent, especially a better match for DAS28. The 95th percentile of predicted PK data through VPC is a slight under-prediction and the CRP model over-predicted drug inhibition at 5th percentile slightly, which will be discussed in the part of discussion. Despite these small deviations, the 90% prediction interval of simulated data covered most of the observations. The bootstrap results are also shown in Table 2. The typical values of parameters and IIV estimates in the final model were pretty close to the median values from bootstrap validation, and fell within 90%CI of bootstrap parameters completely, which indicated high stability and precision of the final model.

Overall, the good performance of GOF, pcVPC plots and bootstrap estimations reconfirmed that the final PK/PD model was adequately developed and the predictive performance was sufficient to capture PK/PD observations.

### Simulations for Phase Ib/IIa Dose Selection

**FIGURE 5:**
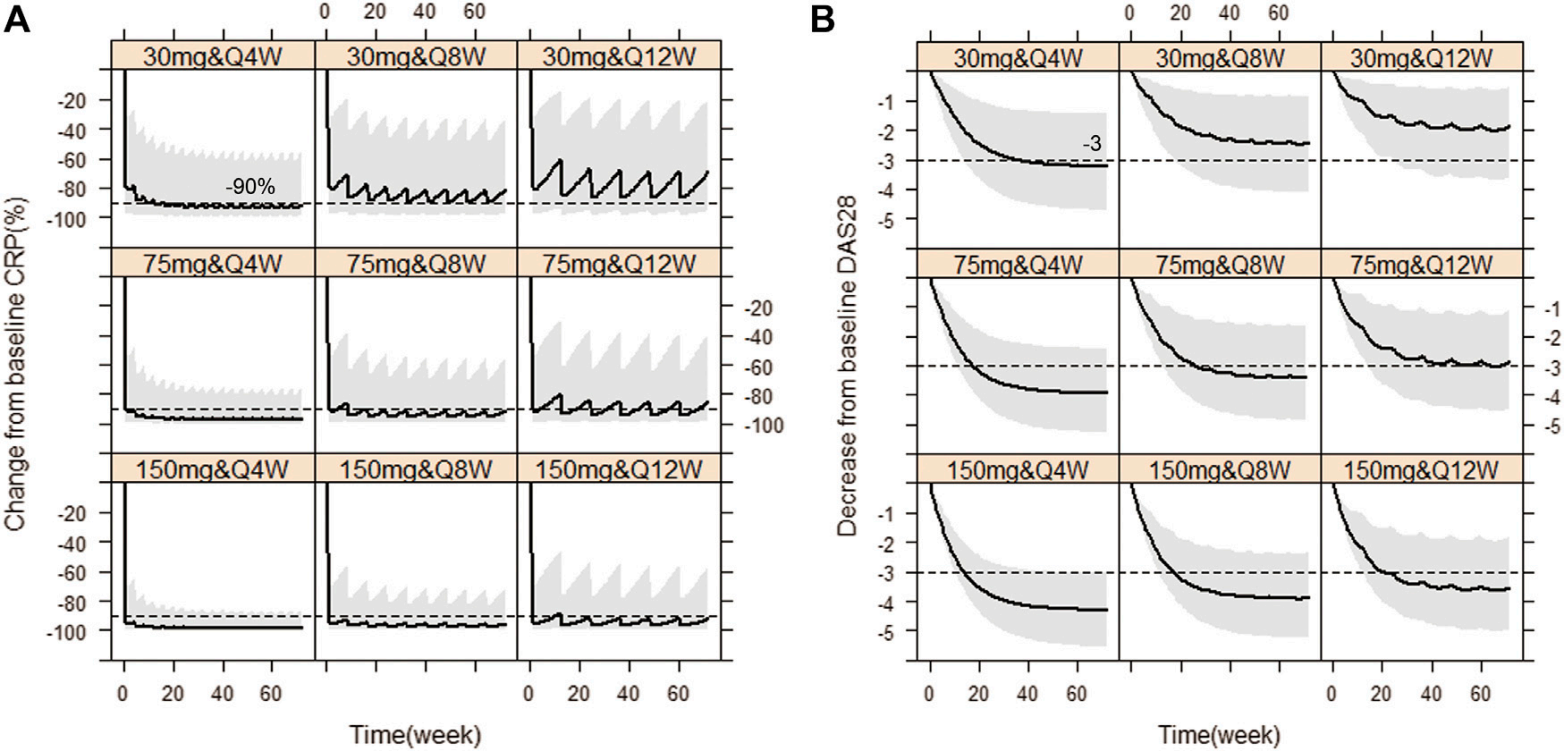
Simulated CRP and DAS28 changes over time under nine dose regimens using final population PK/PD model. The percent change from baseline CRP against time **(A)**, The decrease from baseline DAS28 over time **(B)**. The solid line denotes the 50th percentile of model simulation results and the shaded region presents the 80% prediction interval. Dashed lines show −90% and −3 therapeutic targets for CRP and DAS28, respectively.

Based on the observations, we chose dose levels of 30, 75 and 150 mg as potential maintenance doses so as to rapidly reach the target effect (mean ∆CRP≥90%; ∆DAS28 ≥ 56.5%, i.e. ∆DAS28 ≥ 3 when average baseline DAS28 is 5.3) at steady state, equivalent to tocilizumab (ACTEMRA^®^ HIGHLIGHTS OF PRESCRIBING INFORMATION; U.S. Food and Drug Administration). Then, simulations of those different maintenance dose levels and three varying dose frequencies Q4W, Q8W and Q12W were performed, respectively. Figure 5 showed the Monte-Carlo simulations of CRP and DAS28 changing from baseline over time under different dose regimens using the final PK/PD model. Consistent with observations, the simulation results showed that CRP would decrease very rapidly to the nadir within the first week, while DAS28 changed slowly and reached a plateau after 24 weeks. Multiple dose regimens could decrease the mean CRP levels more than 90% from baseline and reduce DAS28 by more than 3 units, except for 30 mg Q8W, 30 mg Q12W, 75 mg Q12W scenario. The optimal regimens, in terms of achieving mean ∆CRP≥90% and ∆DAS28 ≥ 3, seemed to be 75 and 150 mg as maintenance doses administered every 8 weeks. Approximately 81% and 92% of virtual patients achieved DAS28-ESR <2.6 (the cutoff of RA remission defined by EULAR), when dosed at 75 mg Q8W and 150 mg Q8W, respectively. The 30 mg dose level did not achieve target efficacy when dosed every 8 or 12 weeks. Dosing every 4 weeks was a bit over-dosing and inconvenient to use in clinical practice, however, Q12W dosing frequency resulted in larger fluctuation in efficacy, especially in CRP, and led to a lower proportion of patients nearby target reference line no matter what dose level.

**FIGURE 6:**
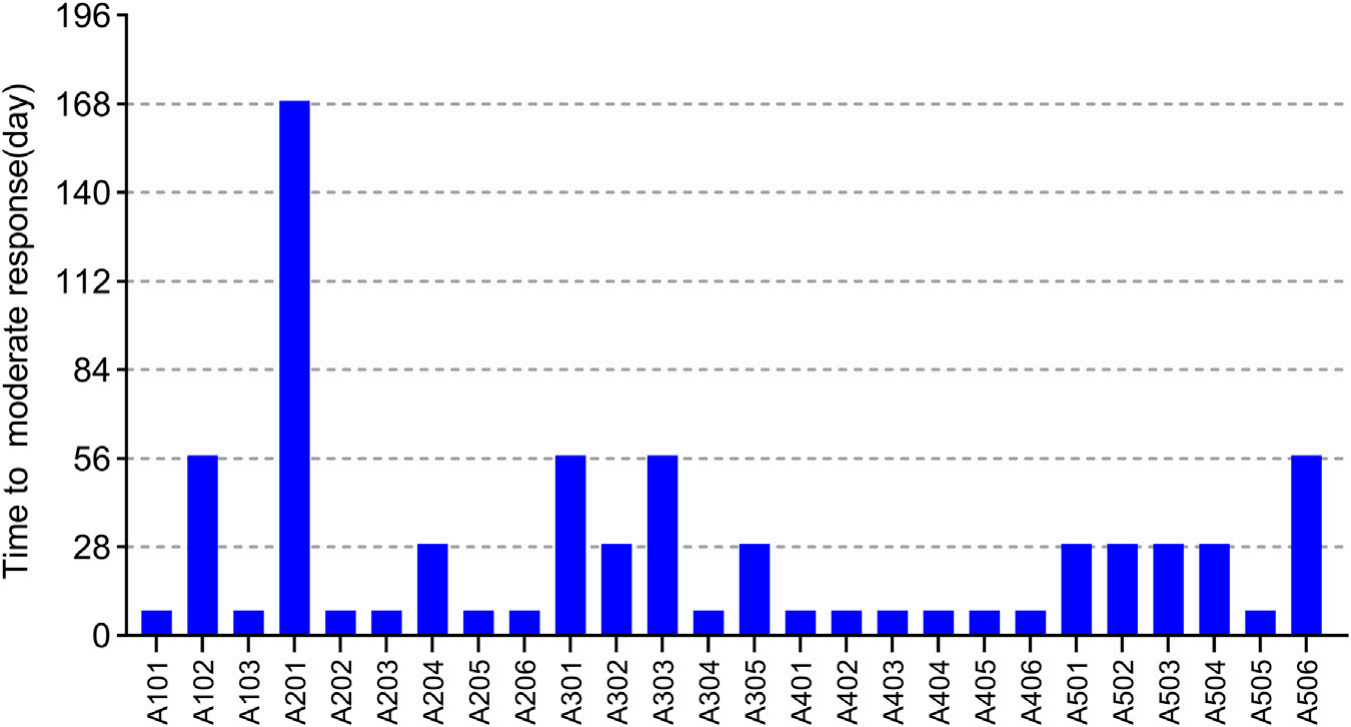
The time required to reach moderate response of 26 subjects in phase Ⅰa trial. Each bar represents one subject.

EULAR (European league against rheumatism) response criteria state (Broeder et al., 2002; Wells et al., 2009) (see Supplementary Table S1): moderate responders were patients with an improvement of >0.6 and a present DAS28 score of <5.1, or an improvement of >1.2 and a present DAS28 score of >5.1. We predefined that once patients start having a moderate response to WBP216, they would begin to feel pain relief intuitively. Patients’ time needed to reach at least moderate response are summarized in Figure 6. The results showed that over half of patients got pain relief within around 10 days, while 12/26 patients did not reach moderate response until at least 28 days from drug administration. WBP216 caused a gradual increase in clinical efficacy during the phase Ⅰa period, which was consistent with the patients’ reports of slow onset of drug action.

**FIGURE 7:**
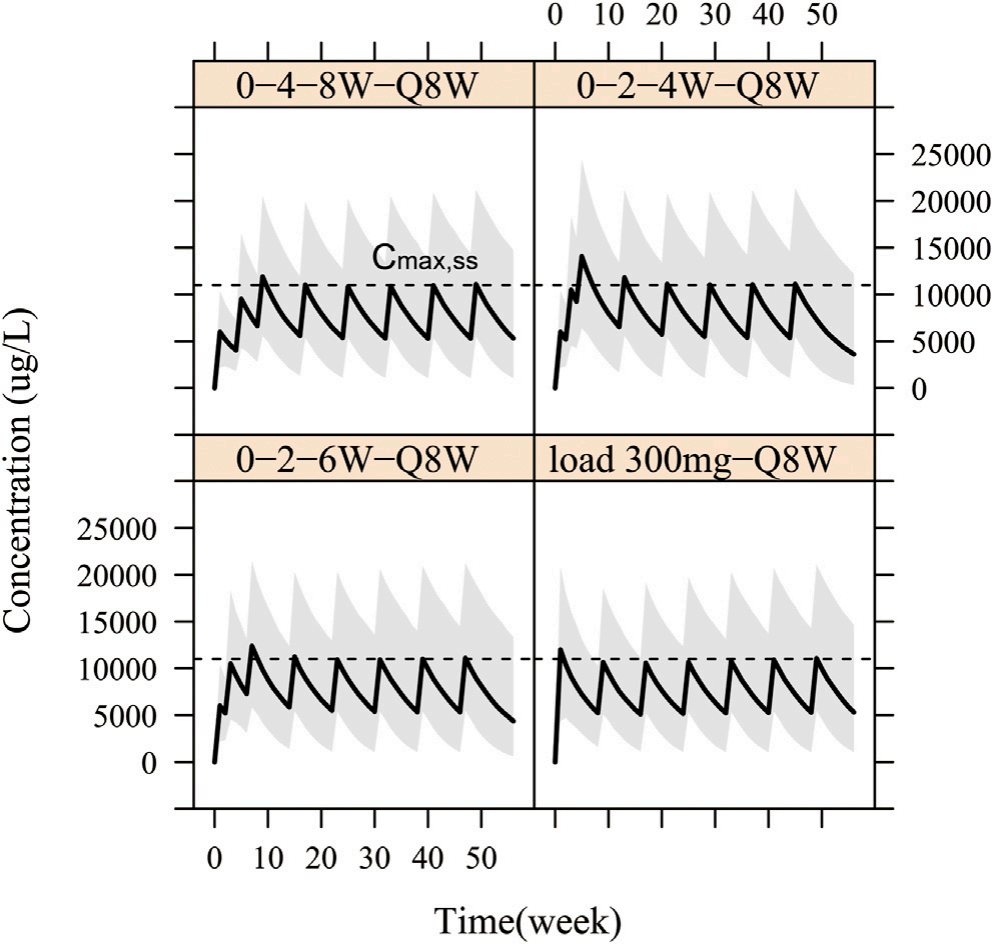
Simulation for pharmacokinetic profiles according to four different loading schedules with 150 mg Q8W as maintenance regimen. The solid line denotes the 50th percentile of model simulation results and the shaded region represents the 80% prediction interval. Dashed lines are maximum concentration at steady state (C_max,ss_).

Therefore, in order to allow patients to benefit from WBP216 therapy more quickly, loading dose regimens were designed. On the basis that the optimal dosing frequency was Q8W during maintenance therapy, we simulated four kinds of “loading dose schedules”, Figure 7 showed examples of varying loading schedules followed by a maintenance dose level of 150 mg Q8W: (1) Loading dose at weeks 0, 4 and 8 followed by Q8W still took more than 8 weeks to reach steady state exposure. (2) Loading dose at weeks 0, 2 and 4 followed by Q8W caused a steep WBP216 concentration increase, exceeding the peak concentration achieved by the maximum dose level (300 mg) in phase Ⅰa, which may raise potential safety concerns. (3) Dosing at weeks 0, 2 and 6 followed by Q8W would reach plateau exposure at the second drug administration. (4) A loading dose of 300 mg, doubling the maintenance dose level, resulted in steady state instantly at the first dosing. Hence, the last two loading dose schedules are highly recommended for future clinical trials of WBP216. The simulated DAS28 profiles under the optimized loading dose regimen (3) and (4) were presented in Supplementary Figure S4, showing that the target efficacy was reached three weeks in advance.

## Discussion

We developed a population PK/PD model that characterized the relationship between WBP216 serum concentrations and the changes in CRP and DAS28. This is the first report of the PK/PD of WBP216 in Chinese RA patients.

Rheumatoid arthritis is a complex autoimmune-mediated inflammatory disease involving both genetic and environmental factors (Smolen et al., 2016). Many factors in RA patients such as the disease process, complications, and concomitant medications could cause significant PK and PD differences between patients and healthy volunteers. Different from some IL-6 (R) antibodies that have been developed PK/PD models based on first-in-human studies with data from healthy volunteers (Xu et al., 2011), our modeling and simulation study in RA patients directly could avoid the aforementioned confounding factors and recommend more accurate dose regimens for phase Ib/IIa trials.

A two-compartment PK model with sequential zero-first order absorption and first order elimination fitted the PK data well. During the process of characterizing PK properties, we found the bioavailability of subcutaneous administration decreased gently with increasing dose levels. This atypical absorption may because large antibodies from the SC site are transported through the tissue interstitium and into the lymphatic system slowly accompanied by tissue metabolism and hydration etc (Richter et al., 2012; Jung et al., 2018). Adopting a base PK model structure with sequential zero-first order absorption (OFV = 4,935) performed significantly better than a traditional two-compartment model with first order absorption rate (OFV = 5,088) or a saturated absorption model of adding the Michaelis–Menten equation to bioavailability (OFV = 5,026). Although the NCA analysis suggested a slight trend of absorption saturation, it seemed not obvious enough to fit a typical M–M equation best in absorption phase. Linear clearance was sufficient to describe WBP216 elimination rather than a linear plus Michaelis–-Menten elimination of tocilizumab (Abdallah et al., 2017), probably because WBP216 binds to free IL-6 while tocilizumab binds the IL-6R involving complex internalization. The typical CL/*F* (0.015 L/h) from model estimates was slightly smaller than CL/*F* of sirukumab (0.019 L/h, corrected by *F*) (Xu et al., 2011), a IL-6 antibody, but the estimated V1/*F* (8.1 L) of WBP216 seemed to be larger than 4.1 L in sirukumab and 5.6 L in tocilizumab. Therefore WBP216 has a longer half-life. PK parameters showed high inter-individual variability, however, only ALT was identified as a covariate accounting for 18% of the variability of CL/F. CL/F was negatively correlated with ALT with exponent −0.833. Chunze Li etc. also reported that clearance of trastuzumab emtansine correlated significantly with baseline albumin and AST (Li et al., 2017). And the clearance of another IL-6 antibody, siltuximab, was found to be impacted negatively by ALT as well in its poplulation PK analyis containing 460 participants (Nikanjam et al., 2019). It is generally accepted that a therapeutic antibody is unlikely to be impacted by functional hepatic impairment (Dirks and Meibohm, 2010). We did not have sufficient data to explain this phenomenon. Dose adjustment for hepatic dysfunction was undetermined, and this would require a large target population to validate this point further. Some studies reported that weight was an important factor affecting CL/F (Abdallah et al., 2017) or V1/F (Li et al., 2018). Our study did not find this covariate, perhaps because of limited sample size with narrow weight range (61.5 ± 7.9 kg) in this phase Ⅰa trial and the stringent covariate entry/elimination criteria set. In addition, only 3/27 patients exhibited ADA positive (Table 1), so ADA was correspondingly identified as a non-significant covariate influencing pharmacokinetic behaviors. All ADA samples of the outlier subject were detected negative, which could not impact ADA conclusion after exclusion.

We did not adopt the strategy of modeling the PK and PD data concurrently because the high variability of CRP or DAS28 would affect the estimate precision of PK parameters. An indirect response model with inhibition on response production was applied for both CRP and DAS28 endpoints based on WBP216’s mechanism of action. Fast decreasing CRP is able to reflect the binding IL-6 ability of WBP216 directly since hepatic production of CRP is mainly mediated by IL-6 (Vermeire et al., 2004), while slow changing DAS28 tracks RA-related clinical efficacy closely. Unlike some early clinical studies that only focused on a fast-decreasing biomarker (i.e., CRP) (Xu et al., 2011; Mayer et al., 2015; Li et al., 2018), we evaluated both fast- and slow-decreasing endpoints simultaneously to understand the efficacy of WBP216 comprehensively. As Table 2 shown, whatever K_in,CRP_ or K_out,CRP_ had greater value than those of DAS28, hinting high turnover rate of CRP. K_in,DAS28_ 0.003 h^−1^ and K_out,DAS28_ 6*10^−4^ h^−1^ were very closed to reported values of tocilizumab (K_in,DAS28_ 0.0037 h^−1^; K_out,DAS28_ 7.2 × 10^−4^ h^−1^) (Bastida et al., 2019). While Levi et al. reported K_in,DAS28_ 0.011 h^−1^ and K_out,DAS28_ 15.8 × 10^−4^ h^−1^ using data from 4 phase III studies of tocilizumab, which was almost 2 fold of our estimated DAS28 parameters (Levi et al., 2013). The differences in above reports may because the DAS28 baseline of subjects was about 5.3 in our and Bastida’s studies instead of 6.8 in Levi’s research. The EC_50,CRP_ (194.37 ug/L) for CRP was fairly smaller than that of DAS28 (1,576.3 ug/L), which indicated a higher concentration was needed for half-maximally decreasing DAS28. Covariates analysis showed that baseline free IL-6 was expected to be positively correlated with K_in,CRP_, since CRP production is mainly stimulated by IL-6 in body. However, no covariates were discovered affecting K_in_ and K_out_ of DAS28. Baseline of free IL-6 was negatively associated with the EC_50_ for CRP. The addition of baseline free IL-6 in model was able to explain 59.2% and 55.6% of IIV for K_in,CRP_ and EC_50, CRP_ respectively. Those limited sample size and narrow demographic or laboratory data may not provide fairly accurate covariate impacts on PK/PD parameters but they provided a reference for future covariate analysis in larger population.

The final population PK/PD model was evaluated by GOF plots, pcVPC and bootstrap. Slight deviations were observed in the 95th percentile of serum concentration and 5th percentile of CRP between respective predicted and prediction-corrected observations (Figure 4). It was noteworthy that one of patients in 300 mg group had extremely high PK exposure, over three fold of other subjects, which raised the 95th percentile of the observed concentration significantly. WBP216 was able to inhibit CRP to a very low level, almost close to zero, while the lower limit of quantitation of CRP can only reach 0.01 mg/L. Our model is expected to have a higher uncertainty near zero because of high IIV and residual errors, so the model predicted a lower CRP 5th percentile value than that of observed data. However, this slight deviation did not affect overall predictive ability since the 90% prediction interval of simulated data covered the majority of the observations.

WBP216 is the IL-6 monoclonal antibody with the longest half-life (40–60 days) by far. By comparison, the half-life of tocilizumab is reported as 11–13days and siltuximab as around 21day, leading to a dosing regimen of once every three or four weeks in clinical practice (ACTEMRA® and SYLVANT^®^, HIGHLIGHTS OF PRESCRIBING INFORMATION; FDA). According to our simulation results (Figure 5), the long half-life of WBP216 would allow it to be optimally administered once every 8 weeks. Dosing every 4 weeks did not offer any advantage since excess drug exposure would occur. Although the simulation results showed 150 mg Q12W seemed also acceptable, the Q8W dosing frequency was able to maintain a more stable change in CRP and DAS28. A score of DAS28-ESR<2.6 defines RA remissions (Anderson et al., 2012; Smolen et al., 2016). In our simulations with the baseline DAS28 around 5.3, when dosing 75 mg Q8W and 150 mg Q8W, approximately 81% and 92% of virtual patients were able to achieve DAS28-ESR <2.6 after 24 weeks therapy, respectively. So both of dose levels (75 and 150 mg) will deserve to be tested in future clinical studies.

A few subjects in phase Ⅰa complained of getting limited relief until one or two months after drug administration (Figure 6). This prompted us to design four loading schedules used for simulation. An initial loading regimen of dosing at weeks 0, 2 and 6, followed by a maintenance regimen of Q8W, achieved steady state at the second administration, which was consist with dosage regimen of infliximab (RENFLEXIS, HIGHLIGHTS OF PRESCRIBING INFORMATION; FDA). Another good loading dose option is to double the maintenance dose level. The simulated DAS28 profiles under the two optimized loading dose regimen did show that three weeks were saved to reach the target efficacy with the maintenance dose of 150 mg Q8W (Supplementary Figure S4).

Although ACR20/50/70 endpoints were also measured in this early clinical study, there was no obvious dose-dependent relationship in the probability of achieving ACR20/50/70 efficacy in this phase Ia study. The combination of both early biomarkers (CRP and DAS28) helped to build the PK/PD relationship. A population PK/PD approach proved again useful in integrating all available PK/PD data during early clinical phases. Our study provided a relatively complete paradigm to accelerate clinical development for similar drugs as well.

The limitations of the model are as follows: (1) Baseline ALT and free IL-6 considered to be statistically significant were observed as covariates. Nevertheless, the limited number of patients in our study and the strict entry criteria set for the clinical trial may result in ambiguous covariate effects. Therefore, the confirmation of covariate effects should be kept in mind in future studies, which will have more data of target population added in. (2) In order to design dose regimen for a MAD study, it was assumed that the PK/PD relationship based on 36 patients in the Phase Ia study lasting 24weeks can be extrapolated to a larger target population and a longer term study. Attention should be paid to this model hypothesis when drawing conclusions from the simulation results. Patients with different disease states, disease progression, drug resistance and combination may invalidate this hypothesis.

In summary, a population PK/PD model was first successfully established for WBP216. Fast-decreasing (CRP) and slow-decreasing (DAS28) biomarkers were modeled concurrently to assess efficacy of WBP216 fully. For WBP216 with an exceptionally long half-life (40–60 days), two kinds of loading dose regimens are recommended for the next clinical studies. We expect that the modeling and simulation will be valuable for dose selection during future clinical trials, and provide a reference for the PK/PD studies of similar antibodies.

## Data Availability

The raw data supporting the conclusions of this article will be made available by correspondence authors, without undue reservation.
