# Supplementary material for: Modeling and Simulation to Support Phase Ib/IIa Dose Selection for WBP216, A Long Half-Life Fully Human Monoclonal Antibody Against Interleukin-6
Source: Front Pharmacol. 2021 Feb 18;12:617265. doi: 10.3389/fphar.2021.617265 (PMC7930490; doi:10.3389/fphar.2021.617265)
Supplement: Supplementary file 1 [file datasheet1.docx]

**Supplementary Material**

**Supplementary Table S1.**  EULAR response criteria

| DAS28 improvement→ | >1.2 | >0.6 and≤1.2 | ≤0.6 |
| --- | --- | --- | --- |
| Present DAS28↓ |  |  |  |
| ≤3.2 | good response | moderate response | no response |
| >3.2 and≤5.1 | moderate response | moderate response | no response |
| >5.1 | moderate response | no response | no response |

**Supplementary Table S2.**  Decrease of OFV after adding covariates into the models when compared with base models.

| Steps | OFV | ΔOFV |
| --- | --- | --- |
| Base PK model | 4935.5 |  |
| ALT added into CL/F | 4920.7 | -14.8 |
| Base CRP model | 5348.9 |  |
| Base Free IL-6 added  on K_in,CRP_ | 5325.3 | -23.6 |
| Base Free IL-6 added on both of K_in,CRP_ and EC_50,CRP_ | 5305.2 | -43.7 |


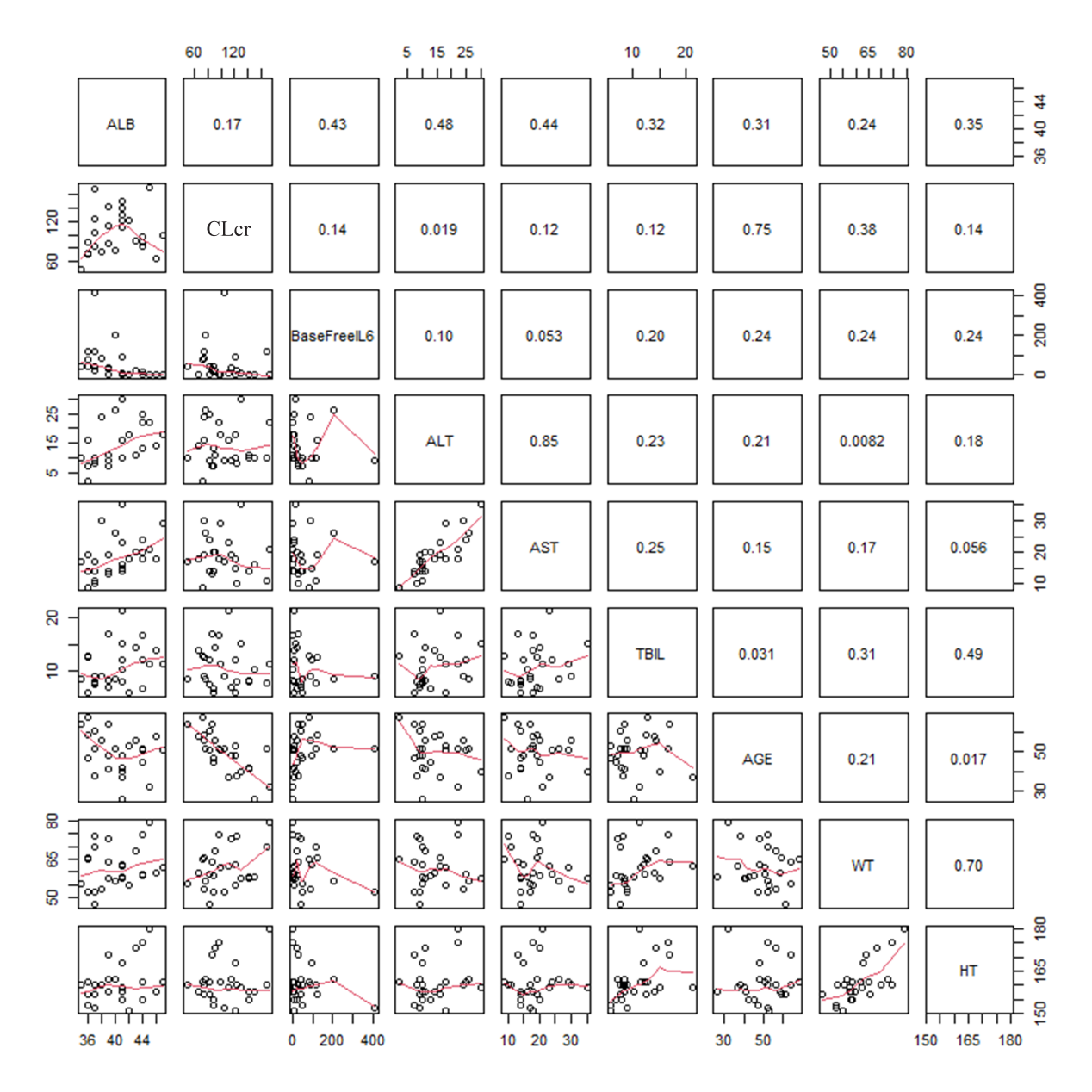


**Supplementary Figure S1.** The correlation diagnosis plot of various covariates. The categorical covariates such as gender and ADA were not included in this plot. The lower panel is the scattered points and fitted smooth curve, and the upper panel is the correlation coefficient. ALB=Albumin, CLcr=Creatinine clearance, BaseFreeIL6=the baseline of free IL-6, ALT=Alanine transaminase, AST=Aspartate transaminase, TBIL=Total bilirubin, WT=Body weight, HT=Hight.

**
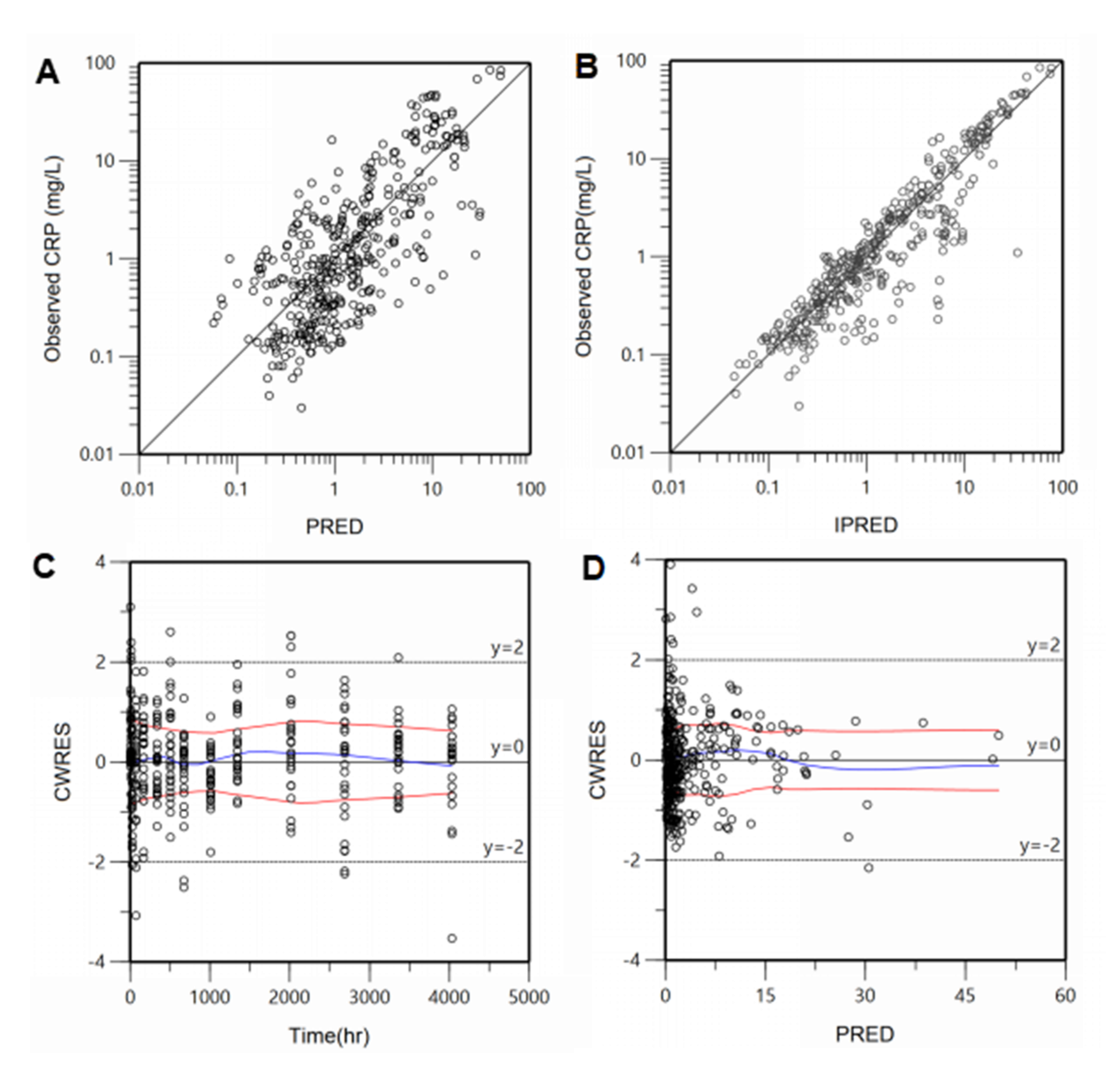
**

**Supplementary Figure S2.** Goodness-of-fit plots for the final population CRP model. Population-predicted CRP versus observed CRP (mg/L) **(A)**; Individual-predicted CRP versus observed CRP **(B)**; The conditional weighted residuals (CWRES) over time after dose (hours) **(C)**; CWRES against population predicted CRP **(D)**. The blue lines are the smoothed LOESS regression lines and the red lines represent LOESS regression to the absolute values of the dependent variable with its negative reflection.


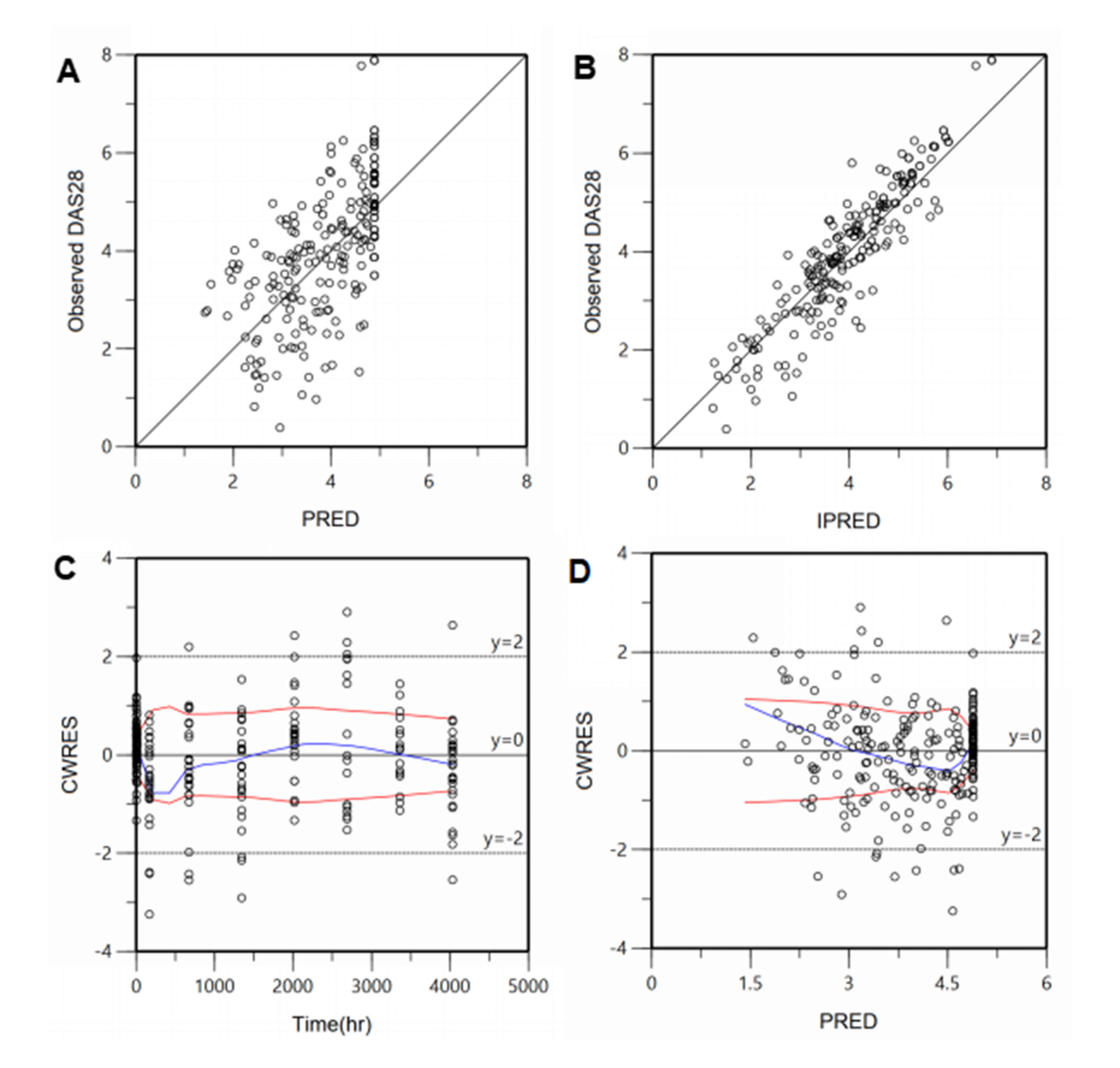


**Supplementary Figure S3.** Goodness-of-fit plots for the final population DAS28 model. Population-predicted DAS28 versus observed DAS28 **(A)**; Individual-predicted DAS28 versus observed DAS28 **(B)**; The conditional weighted residuals (CWRES) over time after dose (hours) **(C)**; CWRES against population predicted DAS28 **(D)**. The blue lines are the smoothed LOESS regression lines and the red lines represent LOESS regression to the absolute values of the dependent variable with its negative reflection.


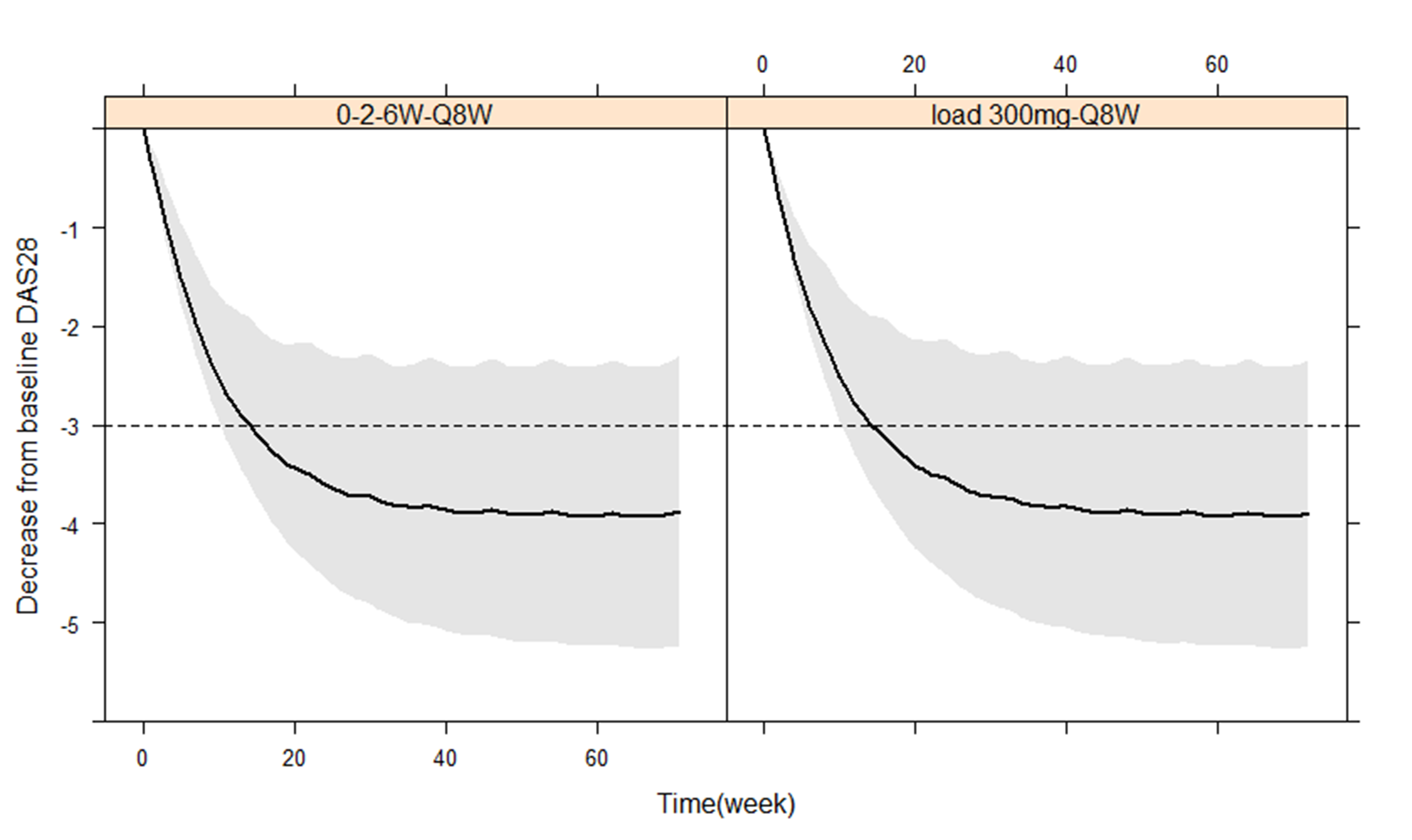


**Supplementary Figure S4**. The simulated DAS28 profiles under the two types of optimized loading dose regimens. The left panel showed the DAS28 profile after dosing at weeks 0, 2 and 6 followed by 150mg Q8W. The right panel presented the situation when giving a loading dose of 300mg, doubling the maintenance dose level (150mg Q8W).
